# Barriers and facilitators to maternal, newborn, and child health service utilization among internally displaced populations in Somalia

**DOI:** 10.3389/fgwh.2026.1880329

**Published:** 2026-07-29

**Authors:** Abdirahman Mohamed Jimale, Mohamed Ahmed Ali, Nor Haji Osman, Aweis Ahmed Moallim, Abdirahman Ahmed Mohamud, Ismail Dahir Ahmed, Abdiweli Mohamed Abdi, Abdikarim Abdi Adam, Osman Mohamed Mohamud

**Affiliations:** 1 Faculty of Health Science, University of Somalia (UNISO), Mogadishu, Somalia; 2 Research Section, Directorate of Health and Human Services, Banaadir Regional Administration, Mogadishu, Somalia; 3 Faculty of Health Science, Al Hayat Medical University, Mogadishu, Somalia; 4 IPC Section, Department of Environmental Health and Climate, Federal Ministry of Health, Mogadishu, Somalia; 5 Faculty of Medicine and Health Sciences, Zamzam University of Science and Technology, Mogadishu, Somalia

**Keywords:** antenatal care, child health, conflict-affected settings, internally displaced persons, maternal health, newborn health, postnatal care, service utilization

## Abstract

**Background:**

Internally displaced populations (IDPs) in Somalia face a disproportionate burden of poor maternal, newborn, and child health (MNCH) outcomes due to conflict, recurrent displacement, poverty, and weak health systems. Evidence on the barriers and facilitators influencing MNCH service utilization in these settings remains fragmented.

**Objective:**

To synthesize the available evidence on barriers and facilitators affecting utilization of MNCH services among IDPs in Somalia, with a focus on antenatal care, skilled delivery, postnatal care, immunization, and care-seeking for childhood illnesses.

**Methods:**

This narrative review examined published literature, policy documents, and humanitarian reports on MNCH service utilization in Somalia and comparable fragile settings. The review focused primarily on sources published between 2015 and 2026, while older sources were retained only when they provided essential conceptual, contextual, or policy information. The review explored both demand-side factors, including socioeconomic hardship, gender norms, and decision-making autonomy, and supply-side factors, including distance, service quality, insecurity, and health system capacity.

**Results:**

The study found that MNCH service utilization among Somali IDPs is constrained by multiple, overlapping barriers. Major barriers include poverty and transport costs, geographic inaccessibility, insecurity, low female autonomy, cultural preferences, shortages of female and skilled health workers, weak referral systems, stock-outs, and limited service readiness. At the same time, important facilitators were identified, including community health workers, women's groups, mobile and outreach services, supportive NGO and government interventions, health system strengthening, and policy frameworks that increasingly recognize IDPs as a priority population. These factors influence service use across the continuum of care and are strongly interrelated.

**Conclusion:**

Improving MNCH service utilization among Somali IDPs requires integrated, equity-focused strategies that combine community engagement, gender-sensitive care, strengthened frontline services, functional referral systems, and better coordination between humanitarian and government actors.

## 1 Introduction

Maternal, newborn, and child health (MNCH) outcomes are fundamental to population health and underpin Sustainable Development Goal 3 targets for reducing maternal and child mortality by 2030 (1). Despite global progress, countries affected by protracted conflict and displacement continue to bear a disproportionate burden of preventable mortality and morbidity among women and children (2). Globally, conflict and fragility disrupt health systems, reduce access to essential services, and contribute to suboptimal maternal and child health outcomes; exposure to armed violence is associated with reduced utilization of antenatal care, institutional delivery, postnatal care, and childhood immunization in Sub-Saharan Africa and other fragile contexts (2–4).

In Somalia, maternal mortality remains alarmingly high, with recent estimates reporting approximately 621–692 maternal deaths per 100,000 live births, among the highest globally. Neonatal and under-five mortality also substantially exceed global averages: Somalia reports roughly 40 neonatal deaths per 1,000 live births and an under-five mortality rate of approximately 101 deaths per 1,000 live births, compared with recent global estimates of approximately 17 neonatal deaths per 1,000 live births and 37 under-five deaths per 1,000 live births (5–8). According to the United Nations Children's Fund (UNICEF), roughly 4 out of every 100 Somali children die within the first month of life and 8 out of every 100 before their first birthday, while approximately 1 in 20 women of reproductive age die from pregnancy-related causes annually (6). Care-seeking for childhood illnesses such as acute respiratory infections and diarrhoea remains low, further exacerbating child health risks (7, 9, 10).

Within this fragile landscape, internally displaced populations (IDPs) represent some of the most vulnerable groups. An estimated 2 million people in Somalia are internally displaced due to conflict, insecurity, and environmental shocks, often residing in overcrowded settlements with limited infrastructure and access to basic services (10). Displacement compounds traditional barriers to care including geographic distance, cost, and sociocultural norms with additional challenges such as insecurity, transient living conditions, and weak health service availability (11). Despite recognition of these issues, systematic evidence on MNCH service utilization specifically among Somali IDP populations is limited and fragmented.

Understanding the barriers and facilitators to maternal, newborn, and child health (MNCH) service utilization in settings of conflict and displacement is essential for informing effective health policy and humanitarian strategies (12, 13). In humanitarian emergencies, women and children experience heightened vulnerability due to reduced access to preventive and curative care, disruption of routine health services, and social marginalization, leading to delayed or forgone care and higher mortality risk (14–16). Despite global and regional efforts to improve MNCH outcomes, evidence suggests that health-seeking behaviours in conflict-affected contexts are influenced by complex individual, household, community, and systemic factors that are not fully understood, particularly among internally displaced populations (2, 11, 17).

Existing evidence from Somalia shows persistently low utilization of essential maternal, newborn, and child health (MNCH) services, including antenatal care, skilled birth attendance, postnatal care, immunization, and care-seeking for common childhood illnesses (18, 19). However, much of the available literature focuses on the general population, a single service area, or broader humanitarian settings, with limited attention to internally displaced populations (IDPs) as a distinct high-risk group. As a result, the specific barriers and enabling factors shaping MNCH service utilization among displaced women and children in Somalia remain insufficiently synthesized. This study addresses that gap by focusing specifically on Somali IDPs and by examining both barriers and facilitators across the continuum of MNCH care. In doing so, it provides a more comprehensive and policy-relevant understanding of healthcare utilization in one of the country's most vulnerable populations.

The primary objective of this narrative review is to synthesize the evidence on factors influencing MNCH service utilization among internally displaced populations in Somalia. Specifically, the review explores barriers and facilitators affecting the uptake of antenatal care, facility delivery with skilled attendance, postnatal care, childhood immunization, and care-seeking for common childhood illnesses. By integrating Somalia-specific evidence with lessons from comparable fragile and displacement-affected settings, the review aims to inform context-sensitive strategies and policies that can improve equitable access to essential MNCH services for displaced women and children.

The scope of this review focuses on maternal, newborn, and child health (MNCH) services and their utilization patterns within displacement contexts, specifically addressing key aspects such as prenatal care services, including the frequency and determinants of antenatal visits; delivery services, particularly facility delivery with skilled birth attendants and the associated determinants; postnatal care, emphasizing access to post-delivery maternal and newborn follow-up care; immunization services, focusing on the uptake and access to routine childhood vaccinations; and child health services, examining care-seeking behaviors for common childhood illnesses and preventive interventions for child health.

The study examines both demand-side factors (e.g., socio-economic status, cultural norms, decision-making autonomy) and supply-side determinants (e.g., distance to facilities, service quality, security) that influence MNCH service utilization among internally displaced populations in Somalia and similar fragile settings.

## 2 Methodology

This study used a narrative review design to synthesize evidence on barriers and facilitators affecting maternal, newborn, and child health (MNCH) service utilization among internally displaced populations (IDPs) in Somalia. A narrative approach was selected because the available evidence is diverse, including peer-reviewed studies, policy documents, and humanitarian reports.

Relevant literature was identified through searches of PubMed, Google Scholar, ScienceDirect, and institutional sources, including WHO, UNICEF, UNFPA, UNHCR, the Somali Ministry of Health, and the Somaliland Ministry of Resettlement and Humanitarian Affairs, particularly the Somaliland Sectoral Displacement Assessment reports. Search terms included combinations of: *maternal health*, *newborn health*, *child health*, *MNCH*, *antenatal care*, *skilled birth attendance*, *postnatal care*, *immunization*, *health service utilization*, *barriers*, *facilitators*, *internally displaced persons*, *IDPs*, *Somalia*, *conflict-affected settings*, and *humanitarian settings*.

Sources were included if they addressed MNCH service utilization among IDPs in Somalia or provided relevant evidence from comparable fragile and displacement-affected settings. Eligible sources included qualitative, quantitative, and mixed-methods studies, reviews, policy documents, and humanitarian reports. Sources were excluded if they were unrelated to MNCH service utilization, did not address displacement or fragile settings, or lacked sufficient information on barriers, facilitators, or health-system context.

The review prioritized literature published between 2015 and 2026, while older sources were used only when they provided important contextual, conceptual, or policy information. Extracted findings were organized thematically into demand-side barriers, supply-side barriers, and enabling factors. Demand-side themes included poverty, transport costs, gender norms, decision-making autonomy, cultural beliefs, and health literacy. Supply-side themes included distance to facilities, insecurity, service availability, quality of care, workforce capacity, referral systems, and availability of medicines and supplies. Facilitators included community-based interventions, mobile and outreach services, NGO and government support, health-system strengthening, digital and data-driven approaches, and policy frameworks. Findings were synthesized narratively because the included sources varied in design, population, and outcomes. Somalia-specific evidence was prioritized, while evidence from comparable fragile settings was used to support interpretation where Somalia-specific evidence was limited. Because this study was designed as a narrative review rather than a systematic review, a prospective screening log was not maintained and exact PRISMA-style counts of records identified, screened, excluded, and included cannot be reported retrospectively without risking inaccurate reporting. To improve transparency, the review instead summarizes the main categories of sources used in the synthesis, including peer-reviewed empirical studies, review and conceptual literature, policy and institutional documents, and humanitarian or UN reports. Somalia-specific and IDP-focused sources were prioritized, while evidence from comparable fragile and displacement-affected settings was used only where Somalia-specific evidence was limited. The principal categories of sources included in the narrative synthesis are summarized in Table 1.

**Table 1:** Summary of source categories included in the narrative review.

Source category	Examples included in the narrative synthesis	Purpose in the review
Peer-reviewed empirical studies	Somalia IDP studies, maternal health studies, vaccination studies, and comparable fragile-setting evidence	To synthesize observed barriers, facilitators, and utilization patterns.
Reviews and conceptual literature	Fragile-setting ANC reviews, conflict-health reviews, and the Three Delays literature	To guide interpretation and organize mechanisms affecting care seeking and service delivery.
Policy and institutional documents	Somali Ministry of Health, EPHS, HSSP, RMNCAH strategy, Community Health Strategy, MRHA/SSDA reports	To align findings with current health-system and displacement policy frameworks.
Humanitarian and UN reports	WHO, UNICEF, UNFPA, UNHCR and humanitarian situation reports	To capture recent displacement, service-delivery, and emergency-response context where peer-reviewed data are limited.

## 3 Evidence synthesis: barriers to MNCH service utilization in Somalia's IDP context

### 3.1 Socioeconomic barriers

Socioeconomic deprivation is one of the most persistent barriers to maternal, newborn, and child health (MNCH) service utilization among internally displaced populations (IDPs) in Somalia. Displacement commonly disrupts livelihoods, weakens household purchasing power, and increases dependence on informal or unstable income sources. In Somalia, poverty is widespread, and recent public health analyses indicate that around seven in ten people live on less than USD 1.90 per day, while recurrent drought, conflict, and displacement continue to undermine food systems and household resilience. For displaced women, this context reduces the ability to pay for consultations, medicines, diagnostic tests, transport, and other indirect costs associated with seeking care, even when some primary services are nominally free. These economic pressures are particularly consequential for antenatal care (ANC), facility delivery, postnatal care (PNC), immunization follow-up, and care seeking for childhood illness, all of which often require repeated contact with health facilities rather than a single visit alone (20, 21).

The Somali literature consistently identifies lack of money as a major barrier to maternal healthcare. A WHO policy brief on maternal and child health in Somalia reported that 65% of women cited lack of money as the main barrier to accessing healthcare during pregnancy, underscoring the central role of affordability in delayed or forgone care (20). The same brief noted that maternal complications can generate catastrophic expenditure because of high out-of-pocket payments, which is particularly relevant in IDP settings where households have minimal savings and weak social protection. Country health profiles also show that Somalia's health financing remains heavily donor dependent and that out-of-pocket spending continues to burden households, indicating that financial risk protection for poor and displaced families remains limited (20).

Studies from Somali IDP settings reinforce these findings at community level. The 2021 qualitative study among IDPs in Mogadishu found that extreme poverty, inability to pay for transport, and limited resources for associated service costs reduced women's use of maternal and child health services. More recently, a 2026 community-based study in Banadir reported that financial constraints remained one of the major barriers to ANC, skilled birth attendance (SBA), and PNC among displaced women. Likewise, evidence on institutional delivery in Somali IDP camps shows that home births are often driven by financial limitations, even where humanitarian actors support some services, suggesting that hidden costs and the broader poverty of displaced households still shape utilization decisions (16, 17, 22).

Economic instability also interacts with displacement itself. IDP families often move repeatedly due to eviction, drought, flooding, or insecurity, making continuity of care difficult. Such mobility may interrupt ANC schedules, child immunization, and postpartum follow-up, while also reducing women's capacity to prioritize preventive care over immediate household survival needs. Recent WHO emergency analyses describe displacement in Somalia as driven mainly by drought and conflict, with overcrowded settlements and food insecurity compounding vulnerability. In practical terms, this means that women may defer care until complications become severe, while children may miss routine vaccination or treatment because care seeking competes with the daily struggle for food, water, and shelter (21).

### 3.2 Cultural, social, and health-literacy barriers

Cultural and social norms strongly influence health-seeking behaviour among Somali IDPs. Gender relations, perceptions of modesty, trust in providers, and family authority structures all affect whether women can seek MNCH care and when. In qualitative research from IDP camps in Mogadishu, women described social expectations that limited their autonomy, especially when husbands or senior relatives controlled decisions about leaving the home, spending money, or choosing where to seek care. This type of constrained decision-making is especially problematic for urgent obstetric care, where delays can rapidly become life threatening (16, 23).

A major gender-based barrier is the preference for female healthcare providers and discomfort with care from male providers. In conservative Somali communities, modesty norms may discourage women from attending ANC, facility delivery, or postnatal services when female staff are unavailable. The 2021 IDP qualitative study identified women's reluctance to be seen by male health workers as an important factor affecting service use, while broader Somali maternal health research also points to sociocultural acceptability as a determinant of where women give birth. In settings already affected by shortages of skilled staff, the absence of female midwives or nurses can therefore directly reduce utilization (16, 23). The midwifery gap is not only a numerical shortage; it is also shaped by institutional barriers to training, deploying, and retaining female midwives in IDP-heavy and insecure areas, including limited training capacity, uneven distribution of schools and clinical placements, insecurity-related mobility constraints, weak incentives for work in peripheral settlements, limited supervision, and dependence on short-term humanitarian financing rather than sustained public-sector workforce planning (16, 23–25).

Women's limited autonomy in household decision-making also constrains service uptake. Evidence from the 2023 qualitative study on vaccination among IDPs in Mogadishu showed that mothers often could not make decisions independently about their children's health and needed support or permission from husbands or mothers-in-law. Although this study focused on childhood vaccination, the same family power dynamics plausibly influence ANC attendance, facility delivery, and PNC, especially in contexts where service use requires travel, spending, or interaction with outsiders. Such patterns are consistent with the broader Somalia literature showing that women's health-seeking is shaped not only by knowledge but by household authority and gendered mobility restrictions (23, 26).

Cultural misconceptions and reliance on traditional practices further affect care seeking. In the Mogadishu IDP qualitative study, participants described trust in traditional birth attendants and home-based practices, particularly when pregnancy and childbirth were viewed as normal events that did not require formal medical attention unless complications developed. Similar patterns have been reported in national Somali studies on maternity care, where poor knowledge of the benefits of facility delivery and low awareness of danger signs were associated with non-use of services. For child health and immunization, the barrier appears to combine limited information with misinformation: some caregivers may not know vaccine schedules or the preventive purpose of repeated doses, while others fear side effects or are influenced by rumours about vaccine safety (16, 23, 24, 26). Distinguishing lack of information from active misconceptions is important because the former requires clear education and reminders, while the latter requires trust-building dialogue through community leaders, women's groups, and trusted health workers (16, 23, 24, 26).

### 3.3 Geographical, physical, and spatial access barriers

Geographical and physical inaccessibility remains a major obstacle to MNCH service utilization in Somalia's displacement settings (16). Many IDP settlements are located on urban peripheries or in poorly served areas where access to formal health facilities is limited. Even when services are geographically present within a district, women may still face long walking distances, insecurity en route, seasonal access constraints, and weak referral linkages (16). These barriers are particularly serious for labour and obstetric emergencies, when delays in reaching care may lead to maternal or newborn death.

The available evidence consistently identifies distance and transport as practical barriers to care. The 2021 qualitative study among Mogadishu IDPs found that women frequently cited long distances to health facilities and lack of affordable transport as reasons for not seeking maternal and child health services. The 2026 Banadir study similarly concluded that distance to facilities remained a major barrier to ANC, SBA, and PNC utilization among women in IDP settlements. In institutional delivery research from Somali IDP camps, women reported distant facilities, urgent labour, lack of transport, and facility closure as reasons for home delivery. Together, these findings suggest that the physical effort and logistics involved in reaching care remain central determinants of service use (16, 17, 22).

Poor infrastructure in and around IDP settlements intensifies these access problems. National and humanitarian assessments describe Somalia's weak transport networks, insecure roads, and poor infrastructure as major constraints on service delivery and healthcare access. Insecurity on roads also complicates movement of people and supplies. For displaced households living in informal settlements, especially those established rapidly after drought or conflict shocks, roads may be unpaved, flood prone, or inaccessible to vehicles. Consequently, even relatively short distances can become substantial barriers for pregnant women, mothers carrying sick children, or families requiring repeated immunization visits (21). Geographic Information Systems (GIS) and spatial analysis could help make these barriers more visible by mapping IDP settlement locations, population density, road accessibility, and travel time relative to BEmONC and CEmONC facilities, outreach sites, and referral points. Such spatial evidence would support better placement of mobile teams, transport vouchers, maternity waiting options, and emergency referral resources (21).

Geographic marginalization also contributes to discontinuity across the continuum of care. Women may attend one ANC visit but fail to return, deliver at home despite prior contact with the health system, or miss early PNC because travel is difficult once labour begins or after childbirth. Emerging qualitative work on Somalia's maternity continuum of care similarly highlights geographic remoteness, limited facilities, and transportation barriers as major reasons why mothers discontinue healthcare services between pregnancy, childbirth, and the postpartum period. In IDP contexts, this fragmentation is likely to be even more pronounced because settlements are often temporary, underserved, and weakly connected to routine primary healthcare infrastructure (22).

### 3.4 Political, security, and coordination barriers

Political instability, armed conflict, and chronic insecurity continue to undermine MNCH service access in Somalia. These conditions shape both the demand-side and the supply side of care. For households, insecurity restricts movement, increases fear, and may force repeated displacement. For health services, conflict damages infrastructure, disrupts staffing, interrupts supply chains, and limits humanitarian access. WHO's 2026 Public Health Situation Analysis for Somalia emphasizes that prolonged conflict and insecurity remain core drivers of humanitarian need, displacement, and service disruption, while recent humanitarian reporting indicates that women and children continue to face heightened exposure to unmet health needs in this fragile environment (21, 27).

Insecurity affects access to care directly by making travel risky. Women in labour, caregivers of sick children, and families seeking immunization may avoid movement if routes are unsafe or if service availability is uncertain. Conflict also affects access indirectly by contributing to displacement into overcrowded settlements where health, nutrition, water, and protection needs accumulate simultaneously. WHO reported that new displacements in Somalia were driven primarily by drought and conflict, and that overcrowded living conditions increased the risk of outbreaks. In such settings, demand for MNCH services rises while service access often becomes more fragile (21).

Another key political barrier is fragmented coordination across the health and humanitarian architecture. Somalia's health system involves federal institutions, federal member states, regional and district authorities, UN agencies, international NGOs, local NGOs, private providers, and diaspora-supported initiatives. Policy analyses have noted weak governance, fragmented programming, and insufficient coordination and integration of reproductive, maternal, newborn, child, and adolescent health activities. Diaspora-led financing and expertise can act as an important facilitator by supporting facilities, specialist care, training, and emergency assistance; however, when not aligned with public stewardship and common standards, such initiatives may also contribute to fragmentation, uneven geographic coverage, and parallel referral pathways. In practice, fragmented coordination can lead to duplicated efforts in some locations, service gaps in others, inconsistent referral systems, and discontinuity between community outreach, fixed facilities, nutrition programs, immunization, and emergency obstetric care. Such fragmentation is particularly harmful for IDPs, whose care pathways often depend on humanitarian actors rather than stable public-sector systems (20, 28).

Funding volatility compounds these coordination challenges. WHO's 2026 analysis reported that severe funding reductions in 2025 forced a major revision of humanitarian targets, leaving many affected populations without assistance. UNICEF and MSF have similarly described large unmet needs for women and children in Somalia, linked to conflict, displacement, and funding shortfalls. For MNCH services, this means outreach may be reduced, mobile teams may stop operating, commodity pipelines may become unstable, and already fragile services in displacement settings may become even less reliable (21, 27).

### 3.5 Health system and workforce barriers

Health system weakness is one of the most important structural barriers to MNCH service utilization in Somalia's IDP context. Even when women wish to seek care, they may encounter limited availability of skilled providers, poor service readiness, stock-outs, overcrowding, disrespectful care, or facilities that cannot manage complications. In Somalia, these weaknesses are longstanding and are magnified in areas serving displaced populations (20).

A severe shortage of skilled health personnel remains a central challenge. Recent Somalia country profiles indicate that the country has only about three doctors, four nurses and nurse-midwives, and 1.5 midwives per 10,000 population as of 2022, with an uneven distribution that favours more secure urban areas. WHO and UNFPA have also highlighted the shortage of midwives and other skilled birth attendants as a major threat to maternal and neonatal survival in Somalia. This shortfall has direct implications for IDP camps and peripheral settlements, where women may not find trained staff for ANC, labour monitoring, emergency obstetric referral, newborn care, or postnatal follow-up (25, 29).

Service readiness is also inadequate. WHO's maternal and child health policy brief reported major shortages in functional health infrastructure and noted that although many facilities report offering ANC, only a limited proportion can provide the full service package. Country profiles further show that many Somali facilities lack basic equipment and essential medicines, while supply shortages are common because of procurement, storage, transportation, and insecurity-related disruptions. The 2025 Somalia country profile noted that health centers had only 8 of 29 essential medicines and primary care units only 4 of 29 on average. In such circumstances, utilization may remain low not simply because women cannot reach care, but because communities lose confidence in care that is unreliable or incomplete (20).

Overcrowding and poor quality of care in facilities serving displaced populations are additional barriers. IDP settlements often concentrate large numbers of vulnerable women and children in a small area, increasing demand on a limited number of facilities. Qualitative evidence from Mogadishu IDP settings describes how poor provider attitudes, weak communication, and limited trust discourage women from returning for care. Emerging studies from Banadir likewise frame limited healthcare access in IDP sites as a result of both structural scarcity and fragile health infrastructure. For maternity care especially, low-quality interactions, lack of privacy, and weak respectful care may discourage use even when facilities are physically accessible (16, 17).

Finally, health system barriers interact with the other barriers rather than operating in isolation. A poor woman living in an insecure settlement may face long distance, no transport, no female provider, and a nearby facility that lacks medicines or skilled staff. This clustering of disadvantages helps explain why MNCH service use in Somali IDP settings remains low across the continuum of care, from ANC to delivery, PNC, immunization, and care for childhood illness. The literature therefore suggests that increasing utilization will require more than health education alone; it will require simultaneous investment in workforce, supplies, financing protection, outreach, referral systems, and accountable coordination in displacement-affected areas.

## 4 Evidence synthesis: facilitators to MNCH service utilization in Somalia's IDP context

### 4.1 Community-based interventions

Community-based interventions are among the most important facilitators of maternal, newborn, and child health (MNCH) service utilization in Somalia's internally displaced persons (IDP) settings. In fragile and displacement-affected environments, facility-based services alone are often insufficient to ensure continuity of care. Community health workers (CHWs), especially Somalia's female health worker models, can reduce the gap between households and formal health facilities by providing health education, identifying pregnant women and sick children early, promoting antenatal care (ANC), supporting vaccination uptake, and facilitating referral to higher levels of care when complications arise. Somalia's recent Community Health Strategy 2025–2029 explicitly positions trained female health workers as the primary link between households and primary health care facilities, reflecting growing national recognition that community platforms are essential for expanding maternal and child health services in hard-to-reach and underserved populations, including IDPs (30, 31).

Evidence from Somalia also shows that supportive community networks can improve demand for MNCH services. In IDP camps, interventions that work through trusted local women's groups and existing social structures have demonstrated measurable benefits. A cluster-randomized trial in Somali IDP camps found that an adapted participatory learning and action approach implemented with indigenous women's groups improved maternal or caregiver knowledge and significantly increased uptake of measles vaccination and completion of the pentavalent vaccine series (32). Related qualitative work showed that community participation and local social structures can help address misinformation, improve trust in services, and create peer support for care-seeking (23). Although these studies focused strongly on child vaccination, their implications extend across the MNCH continuum, since the same mechanisms of trust, social encouragement, and locally rooted dialogue are highly relevant to ANC attendance, skilled delivery, and postnatal follow-up in displaced communities (23, 32).

### 4.2 Government, NGO, and diaspora support

Government and NGO support remains a major facilitator of MNCH utilization in Somalia's IDP context, particularly where routine public services are weak or interrupted by conflict, displacement, and underfunding. Humanitarian agencies have played a central role in extending care through mobile clinics, outreach teams, fixed maternal and child health (MCH) sites, and vaccination campaigns in settlements where displaced populations face limited access to static facilities. UNICEF's pro-equity immunization analysis for eastern and southern Africa documents that fixed and mobile health facilities in IDP camps, together with outreach teams in insecure and hard-to-reach areas, increased access to and uptake of immunization services among displaced children in Somalia (33). More recent humanitarian reporting from UNICEF also shows that partner-supported MCH and nutrition service points continue to function as important delivery platforms for women and children affected by displacement and fragility (27).

A further facilitator is improving coordination between international agencies, Somali authorities, and local communities. Current Somali health-sector reforms increasingly emphasize harmonization rather than fragmented project-based delivery. The EPHS Implementation Strategy and the new Community Health Strategy both stress coordinated planning, accountability, and stronger links between community services, facilities, and referrals (30, 31). At system level, the Somalia Investment Case also recognizes that NGOs and other non-state actors are indispensable for expanding equitable access to essential services, especially when working under government stewardship and common implementation frameworks (28). In practice, such coordination can help reduce duplication, expand geographic coverage, strengthen referrals for obstetric emergencies, and improve continuity between outreach, immunization, nutrition, and routine MNCH care in IDP settlements (28, 31).

### 4.3 Cultural and social facilitators

Cultural and social factors can also act as facilitators when community attitudes begin to shift in favor of formal healthcare. In Somalia, these positive shifts are often gradual rather than uniform, but the literature suggests that repeated exposure to health education, counseling, and community dialogue can improve acceptance of ANC, childhood vaccination, and birth spacing. The national RMNCAH strategy includes maternal care, essential newborn care, and counseling on birth spacing within its service agenda, reflecting policy-level normalization of these services as part of routine family wellbeing rather than exceptional or culturally foreign interventions (34). At community level, participatory interventions in IDP camps have shown that better knowledge and discussion within trusted women's networks can translate into improved uptake of preventive child health services (23, 32).

Women's empowerment is another important facilitator. Where women have greater decision-making space, stronger social support, and better access to information, MNCH utilization tends to improve. Recent Somalia-based evidence shows that interventions combining community engagement with economic support can increase ANC and skilled birth attendance, suggesting that women are more likely to use services when both social and financial constraints are reduced (35). Similarly, current qualitative work on the maternity continuum of care in Somalia indicates that lack of maternal health education contributes to delayed decisions and underuse of services; by implication, improved knowledge, stronger family support, and more favorable community attitudes are likely to facilitate timely use of care (22). Thus, in Somalia's IDP settings, cultural change should be understood not as a sudden abandonment of traditional norms, but as an incremental process in which women, families, community leaders, and peer networks increasingly recognize the value of preventive and facility-based MNCH care (22, 35).

### 4.4 Health system, digital, and data-system improvements

Health system strengthening is a foundational facilitator of MNCH utilization in displacement settings. Demand for services is unlikely to rise sustainably unless services are available, acceptable, and functional. Somalia's EPHS 2020 and the EPHS Implementation Strategy both emphasize bringing essential services closer to communities, prioritizing women, children, and IDPs, and strengthening the continuum between community-based services and facility-based care (31). These reforms are especially important in IDP settings, where women often require repeated contacts for ANC, delivery, PNC, immunization, nutrition, and management of childhood illness. Improving infrastructure, increasing the availability of medicines and supplies, and building referral systems can therefore make service use more feasible and more worthwhile from the user's perspective (31).

Capacity-building is equally important. Somalia's current health policy architecture recognizes the need to expand and professionalize the health workforce, including community-based cadres and frontline maternal and child health providers (28, 30, 31). Training local workers and harmonizing community cadres under nationally recognized models can improve service quality, trust, accountability, and continuity of care (28, 30, 31). Recent health-system reforms also stress stronger engagement of non-state providers under public stewardship, allowing services in IDP, rural, urban, and nomadic settings to be adapted to context while still aligning with national standards (28). This is particularly relevant in Somalia, where many displaced populations access care through NGO-supported, diaspora-supported, or mixed-delivery arrangements rather than through fully public facilities alone.

Integration of displaced populations into the national health system is another key facilitator. Somalia's EPHS explicitly identifies IDPs as a priority population and frames equitable access as a core principle of universal health coverage (31). This policy orientation matters because it shifts service provision away from a narrow emergency logic toward a more sustained system approach in which displaced women and children are recognized as legitimate users of essential national health services. When paired with outreach, referral strengthening, coordinated contracting or service delivery, and digital health information systems, this integration can improve continuity of MNCH services and reduce the risk that IDPs are served only intermittently through short-term humanitarian interventions (28, 31). Predictive modelling and routine data analytics could further act as facilitators by using facility utilization, stock levels, referral delays, displacement trends, rainfall or drought alerts, and camp population changes to anticipate service bottlenecks before they contribute to maternal or newborn deaths (28, 31). In projects such as Damal Caafimaad and broader digital health reforms, these tools could support early warning for medicine stock-outs, identify facilities at risk of overload, prioritize mobile outreach, and guide targeted supervision where ANC, delivery, PNC, or immunization dropout is rising (36).

### 4.5 Policy and legal frameworks

Policy and legal frameworks provide an important enabling environment for improving MNCH utilization among IDPs in Somalia. Somalia's health policy direction increasingly reflects national and international commitments to equity, universal health coverage, and protection of vulnerable populations. The EPHS 2020 states that essential health and nutrition services should be delivered as close to families and communities as possible, with particular attention to mothers, children, and internally displaced persons (31). The Health Sector Strategic Plan 2022–2026 and the Somalia Investment Case 2022–2027 further reinforce this agenda by prioritizing equitable access, workforce reform, supply systems, stewardship, and collaboration with non-state providers to expand coverage in underserved settings (28, 37).

Somalia's broader displacement policy framework also supports this direction. The National Policy on Refugee-Returnees and Internally Displaced Persons affirms that IDPs should enjoy the same rights and access to services as other citizens, and that durable solutions should be linked to equal access to systems and services (38, 39). Although implementation remains uneven, this principle is highly relevant for MNCH because it provides a legal and normative basis for including displaced women and children in national service planning rather than treating them as permanently separate humanitarian beneficiaries. In parallel, Somalia's RMNCAH strategy and Community Health Strategy embed maternal, newborn, child health, immunization outreach, and community-based service delivery within national priorities (30, 34). Together, these frameworks support the inclusion of MNCH services in both health-sector planning and humanitarian response, creating policy space for more coherent and equitable service delivery to IDPs.

## 5 Discussion

### 5.1 Synthesis of barriers and facilitators

This review shows that barriers and facilitators to maternal, newborn, and child health (MNCH) service utilization among internally displaced populations (IDPs) in Somalia are deeply interconnected rather than operating independently. The findings can be interpreted through the Thaddeus and Maine Three Delays Model: cultural norms, low autonomy, misinformation, and financial hardship primarily contribute to Phase 1 delays in deciding to seek care; distance, transport barriers, insecurity, and weak referral pathways contribute to Phase 2 delays in reaching care; and shortages of skilled staff, absence of female providers, stock-outs, poor communication, and limited emergency readiness contribute to Phase 3 delays in receiving adequate care after arrival (12, 20, 40). In Somalia, current health-sector documents and recent evaluations continue to describe a fragmented and under-resourced health system that relies heavily on humanitarian delivery mechanisms, particularly in hard-to-reach and displacement-affected areas (31, 40).

The facilitators identified in this review are similarly interrelated. Community health workers, women's groups, mobile and outreach services, and stronger coordination between government and humanitarian actors appear to improve service utilization because they address both demand-side and supply-side barriers at the same time. In Somalia, the new Community Health Strategy 2025–2029 positions community-based workers as a bridge between households and primary care facilities, while the EPHS Implementation Strategy emphasizes linking humanitarian services with national service delivery arrangements (31). In parallel, studies from Somali IDP camps show that participatory women's groups and community-based approaches can improve maternal knowledge, trust, and uptake of child vaccination, demonstrating that social support and local participation can strengthen healthcare utilization in fragile settings (23, 32).

The findings also suggest that MNCH use in Somalia's IDP context is best understood through a continuum-of-care perspective. Barriers encountered at one point in care can influence service uptake later in pregnancy and after childbirth. For example, missing ANC because of cost, distance, or low decision-making power may reduce the likelihood of facility delivery and later PNC. Conversely, early contact with trusted outreach workers may increase the chances of timely ANC attendance, birth preparedness, referral acceptance, and follow-up for newborn and child health services (22, 32). This reinforces the need for interventions that connect household outreach, community engagement, facility quality, and referral capacity rather than addressing each MNCH contact separately.

### 5.2 Comparison with global trends

The Somali findings are broadly consistent with evidence from other conflict- and displacement-affected settings, especially Yemen, South Sudan, and Syrian displacement contexts. Across these settings, common barriers include insecurity, damaged infrastructure, shortages of trained staff, out-of-pocket costs, transport problems, and social constraints on women's mobility and decision-making (12, 41–44). A systematic review of antenatal care utilization in 37 fragile and conflict-affected settings found that socioeconomic disadvantage, distance to services, poor quality of care, limited education, and gendered household power relations were among the most frequent determinants of low ANC use, which closely mirrors the patterns identified in Somalia (12).

Yemen shows particularly strong similarities to Somalia. A Conflict and Health case study found that RMNCH service delivery during conflict depended heavily on humanitarian actors, mobile clinics, outreach platforms, and community-based workers because routine facility services had been weakened by insecurity, poor governance, and workforce shortages (41). This resembles Somalia, where NGOs and UN agencies remain central to service delivery in many displacement-affected areas, but where recent policy reforms are trying to align these efforts more closely with national health strategies and essential service packages (31).

South Sudan provides another close comparison. Evidence from Upper Nile and Unity states showed that maternal and child health service provision was constrained by conflict, staffing shortages, weak infrastructure, insecurity, and dependence on humanitarian agencies, while community engagement and coordination mechanisms were important facilitators of service delivery (42). As in Somalia, the lesson from South Sudan is that humanitarian outreach can improve access, but sustainable gains require stronger public stewardship, functioning referral systems, and better integration of emergency and routine services (40, 42).

Findings from Syrian displacement settings also reinforce the Somali picture. Research among Syrian refugee women in Lebanon found that antenatal care coverage was lower among women living in insecure accommodation and among those facing structural and access-related vulnerabilities (44). More recent work from Türkiye showed that crises such as the COVID-19 pandemic can further reduce access to reproductive and maternal healthcare among refugee women, especially when social and household constraints persist (43). Together, these comparisons suggest that Somalia follows a broader global pattern in which displacement magnifies pre-existing barriers to maternal and child healthcare, while community outreach, female frontline workers, mobile services, and better coordination consistently emerge as the most practical facilitators in fragile settings.

### 5.3 Policy and practical implications

The main policy implication is that improving MNCH service utilization among Somali IDPs requires a combined strategy that addresses affordability, proximity, trust, and service quality at the same time. Policymakers should prioritize implementation of existing national frameworks, especially the Health Sector Strategic Plan, EPHS 2020, EPHS Implementation Strategy, and Community Health Strategy, with explicit focus on IDP settlements and other underserved populations (31, 37). These frameworks already provide a basis for equitable service delivery, but their impact will depend on financing, operational coordination, and accountability at federal, state, district, and community levels.

For healthcare providers and health managers, continuity of care should be a central operational goal. ANC, skilled birth attendance, PNC, immunization, nutrition, and treatment of childhood illnesses should be linked through functional referral pathways, routine follow-up, and outreach by trusted frontline workers, especially female providers where possible (20, 31). Recent Somali policy briefs also stress that weak skilled birth attendance, fragmented maternal and newborn care, and poor quality of intrapartum and postnatal care remain major contributors to preventable mortality, indicating that increasing utilization must go hand in hand with improving the quality and readiness of services (20).

For humanitarian organizations and NGOs, the evidence supports maintaining mobile clinics, outreach teams, vaccination campaigns, and community-based engagement in IDP sites, but with stronger alignment to national systems and local governance structures (31, 41, 42). Humanitarian actors should avoid creating parallel delivery models that disappear when funding declines. Instead, they should support integrated service packages, harmonized training, shared referral systems, and local data systems that can follow population movement and changing settlement patterns (31, 40, 42). Programs that also engage husbands, mothers-in-law, community leaders, and women's groups are likely to be particularly important in Somalia, because health-seeking behavior is often shaped by collective decision-making rather than individual preference alone (12, 22, 23).

### 5.4 Gaps in research

Much of the available literature on MNCH utilization among Somali IDPs is cross-sectional, qualitative, or focused on a single component such as immunization or ANC. There is still limited longitudinal evidence tracking women and children over time to understand how repeated displacement, eviction, seasonality, insecurity, and changing camp conditions affect continuity across ANC, delivery, PNC, and child health services. Longitudinal cohort studies and linked routine data systems would be especially valuable for identifying where dropout occurs along the continuum of care and how humanitarian and policy interventions influence service use over time. Such systems should distinguish settled urban IDPs from newly displaced pastoralist or nomadic households, because these groups may differ in mobility patterns, service familiarity, livelihood disruption, and exposure to eviction or seasonal shocks.

Existing Somali studies provide useful evidence on community participation and maternity care gaps, but fewer studies compare which combinations of interventions are most effective in increasing service utilization in IDP settings, such as community health workers, transport support, mobile clinics, digital reminders, women's groups, or facility strengthening. Comparative studies examining effectiveness, cost, scalability, and sustainability would be highly useful for both policymakers and humanitarian actors. Finally, future research should better capture heterogeneity within displaced populations. IDP women are often treated as a single category, yet utilization likely differs by age, parity, marital status, disability, clan or minority status, livelihood security, registration status, and duration of displacement. More disaggregated and mixed-methods research is needed to identify which subgroups are most excluded from essential MNCH services and whether current health-sector reforms are reducing inequities in practice or mainly at policy level.

## 6 Conclusion

Improving MNCH outcomes in Somalia's IDP settings will require more than expanding services alone. It demands a coordinated and equity-focused response that strengthens frontline care, empowers communities, improves referral and continuity of care, integrates displaced populations into national health strategies, and builds longitudinal data systems capable of tracking care dropout across pregnancy, childbirth, the postnatal period, immunization, and childhood illness episodes. By synthesizing fragmented evidence specifically on Somali IDPs, this review adds value by organizing barriers and facilitators across the MNCH continuum of care and interpreting them through displacement-sensitive health-system and Three Delays perspectives. Its contribution lies in moving beyond a descriptive list of known barriers to highlight practical entry points for integrated, data-driven, and IDP-sensitive MNCH service planning in Somalia. Without such longitudinal tracking, it will remain difficult to determine where displaced women and children are lost along the continuum of care or to measure whether policy and humanitarian investments are reducing inequities over time.
